# Supplementary material for: Influence of Beam Angle on Normal Tissue Complication Probability of Knowledge-Based Head and Neck Cancer Proton Planning
Source: Cancers (Basel). 2022 Jun 9;14(12):2849. doi: 10.3390/cancers14122849 (PMC9221467; doi:10.3390/cancers14122849)
Supplement: Supplementary file 1 [file cancers-14-02849-s001.zip › Supplementary_Material_S1.pdf]

Supplementary Material S1: NTCP Equation and Coefficients

Normal tissue complication probability (NTCP) was calculated as $NTCP = \frac{1}{1+e^{-S}}$, where $S = \beta_0 + \Sigma_i(\beta_i \cdot x_i)$ [26]. The coefficients β for each dose value x are presented in Table A1 below. The primary tumor location for all patients was assumed to be in pharynx.

Table S1: Variable coefficients used to estimate the NTCP values [26].

VARIABLES	ENDPOINTS			
	Xerostomia after 6 months		Dysphagia after 6 months	
	Grade ≥ 2	Grade ≥ 3	Grade ≥ 2	Grade ≥ 3
Constant (β_0)	-2.2951	-3.7286	-4.0536	-7.6174
$\sqrt{D_{mean}}(I.Parotid) + \sqrt{D_{mean}}(C.Parotid)$	0.0996	0.0855		
$D_{mean}(Both\ Submandibulars)$	0.0182	0.0156		
$D_{mean}(Oral\ Cavity)$			0.0300	0.0259
$D_{mean}(PCM\ Sup)$			0.0236	0.0203
$D_{mean}(PCM\ Med)$			0.0095	0.0303
$D_{mean}(PCM\ Inf)$			0.0133	0.0341
Primary Tumor Location in Pharynx			-0.6281	0.0387
