# Supplementary material for: Influence of Beam Angle on Normal Tissue Complication Probability of Knowledge-Based Head and Neck Cancer Proton Planning
Source: Cancers (Basel). 2022 Jun 9;14(12):2849. doi: 10.3390/cancers14122849 (PMC9221467; doi:10.3390/cancers14122849)

Supplementary Material S2: OAR mean doses for default- and altered angle plans

Figure S1: Mean doses for all OAR for both default- and altered-angle plans (a,c), and the corresponding differences (b,d, mean dose of default-angle plans minus mean dose of altered-angle plans). OARs in a,b were the target of AIO process and OARs in c,d were optimized only according to the RapidPlan-generated objectives. Dark line in the middle of the box indicates the median, the top/bottom of the box indicates 75th/25th percentile, and the whiskers indicate the range of the data. PCM Inf/Med/Sup = inferior/middle/superior pharyngeal constrictor muscle; Parotid I/C = ipsi-/contralateral parotid gland; Subm I/C = ipsi-/contralateral submandibular gland.

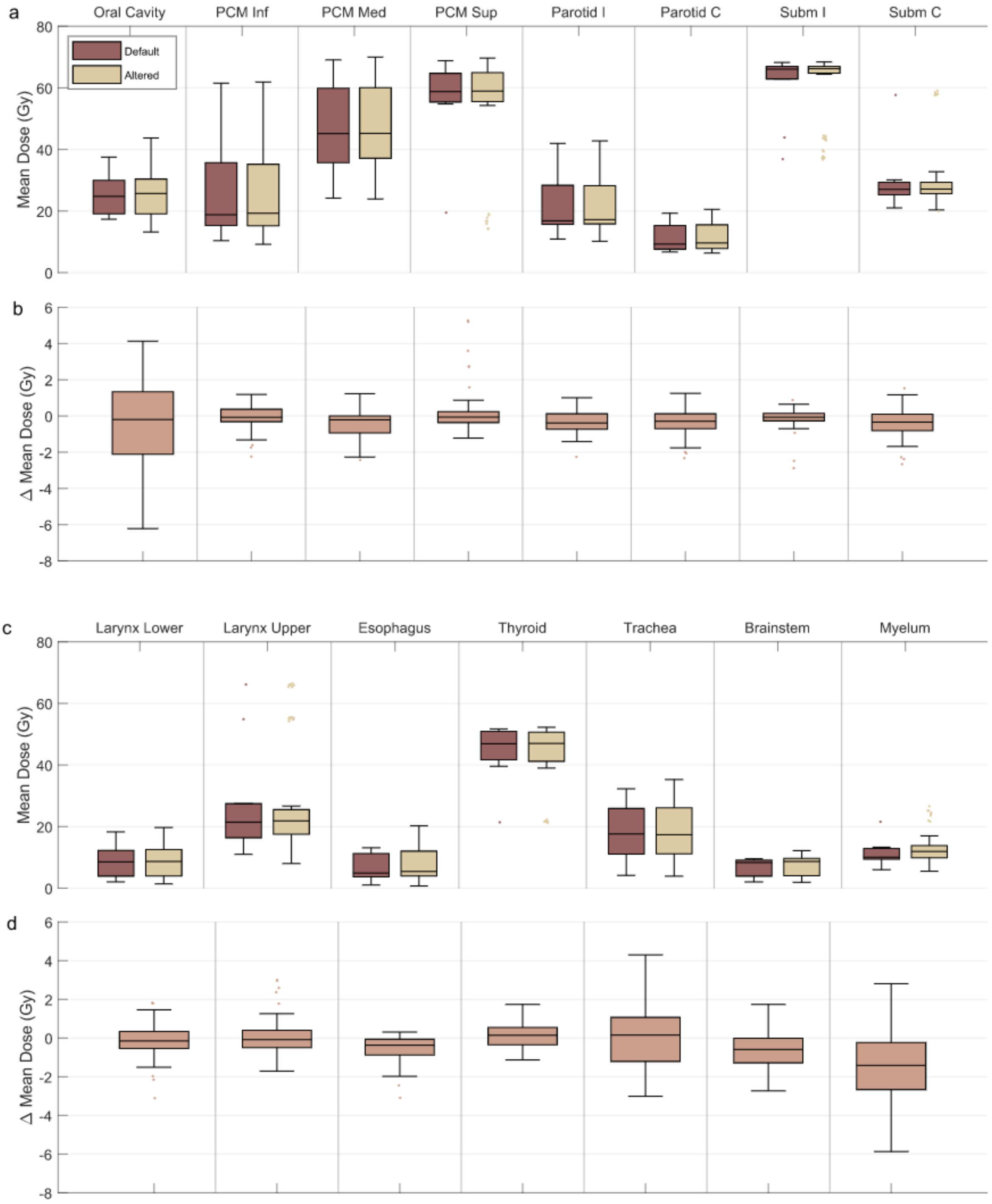

Supplement: Supplementary file 1 [file cancers-14-02849-s001.zip › Supplementary_Material_S2.pdf]
