# Supplementary material for: Influence of Beam Angle on Normal Tissue Complication Probability of Knowledge-Based Head and Neck Cancer Proton Planning
Source: Cancers (Basel). 2022 Jun 9;14(12):2849. doi: 10.3390/cancers14122849 (PMC9221467; doi:10.3390/cancers14122849)

Supplementary Material S3: Delineations of two patients

Figure S2: Delineations of patients 6 and 5. PCM Inf/Med/Sup = inferior/middle/superior pharyngeal constrictor muscle; Parotid L/R = left/right parotid gland; Subm L/R = left/right submandibular gland.

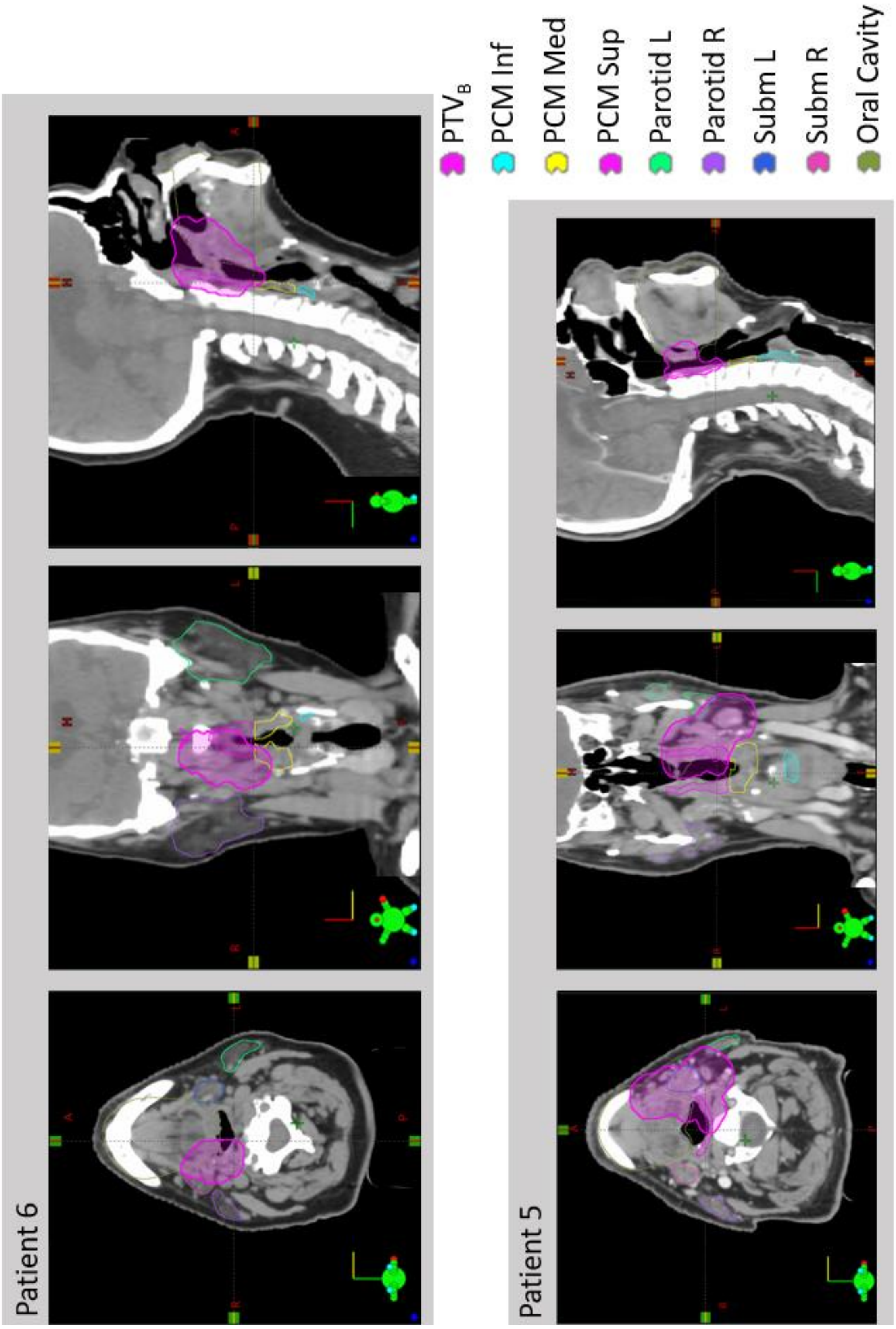

Supplement: Supplementary file 1 [file cancers-14-02849-s001.zip › Supplementary_Material_S3.pdf]
