# Supplementary material for: Influence of Beam Angle on Normal Tissue Complication Probability of Knowledge-Based Head and Neck Cancer Proton Planning
Source: Cancers (Basel). 2022 Jun 9;14(12):2849. doi: 10.3390/cancers14122849 (PMC9221467; doi:10.3390/cancers14122849)

Supplementary Material S4: Results for multiple fields

Figure S3: NTCP$_\Sigma$ and HI over 3/4/5 fields for 3 patients before and after AIO processing.

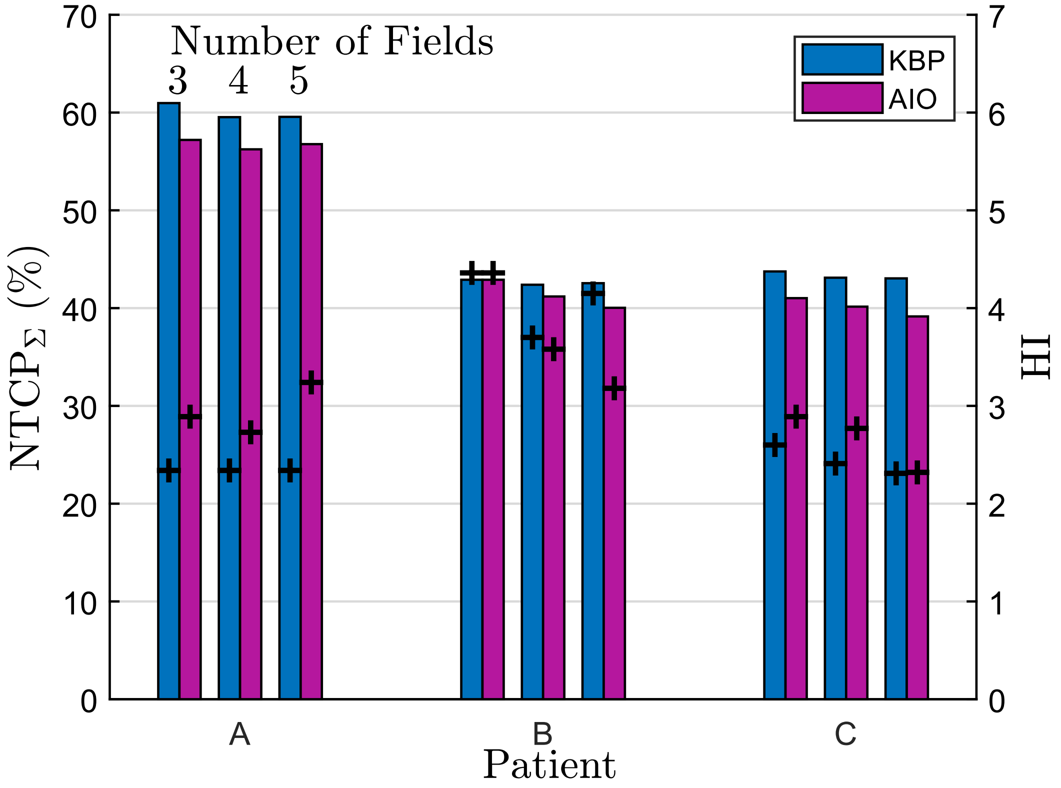

Supplement: Supplementary file 1 [file cancers-14-02849-s001.zip › Supplementary_Material_S4.pdf]
